# Cocultured porcine granulosa cells respond to excess non-esterified fatty acids during in vitro maturation

**DOI:** 10.1186/s13048-021-00904-y

**Published:** 2021-10-28

**Authors:** Meihong Shi, Marc-André Sirard

**Affiliations:** Centre de recherche en reproduction, développement et santé intergénérationnelle, Faculté des Sciences de l’Agriculture et de l’Alimentation, Département des Sciences Animales, Pavillon Institut sur la Nutrition et les Aliments Fonctionnels, Université Laval, Québec, Québec Canada

**Keywords:** NEFAs, Granulosa cells, IVM, Transcriptome

## Abstract

**Background:**

Non-esterified fatty acids (NEFAs) are one of the main lipid components of follicular fluid at concentrations that depend on circulating levels. Elevated levels of NEFAs impair oocyte quality, development potential, and may subsequently influence the metabolism and reproductive fitness of offspring. Granulosa cells (GCs) are the follicular cells that are closely communicating with the oocyte. However, the responses of GCs exposed to high levels of NEFAs when cocultured with cumulus-oocyte complexes (COCs), and how they attenuate the negative effects of NEFAs on oocytes, are unclear.

**Results:**

To better understand this protective effect, monolayers of porcine GCs were cocultured with COCs during *in vitro* maturation (IVM) in the presence of elevated levels of NEFAs. Genomic expression analysis was conducted to explore the responses of the GCs to the elevated levels of NEFAs. After limma algorithm analysis, 1,013 genes were differentially expressed between GCs cultured with and without elevated NEFAs. Among them, 438 genes were upregulated and 575 were downregulated. The differentially expressed genes were enriched in pathways related to metabolism, inflammation, and epithelial-mesenchymal transition.

**Conclusions:**

The pathways and upstream regulators suggested that the cocultured GCs responded to the elevated NEFAs with (1) inhibition of the transition from granulosa to luteal cell, (2) interactions of metabolism change, anti-inflammation, mitochondrial function, and cell transition, (3) intercommunication with cocultured COCs of anti-inflammatory factors.

**Supplementary Information:**

The online version contains supplementary material available at 10.1186/s13048-021-00904-y.

## Background

In mammals, especially in bovine and humans, the contexts of negative energy balance and obesity result in elevated levels of circulating non-esterified fatty acids (NEFAs) and may affect fertility [1, 2]. The elevated NEFAs in circulation originate mainly from lipolysis in adipocytes and from the diet [3]. *In vitro* studies showed that increased NEFA levels can affect follicular growth through impaired viability and steroidogenesis of granulosa cells (GCs) [4, 5]. Moreover, the early presence of high NEFA levels in the follicular fluid can result in subnormal corpus luteum function [6]. High levels of NEFAs during oocyte maturation and early embryo development have hazardous effects on development competence by affecting metabolism and cell fate [3]. In our previous study, exposure to elevated NEFAs level during IVM impaired the development of porcine blastocysts by increasing endoplasmic reticulum (ER) stress, reactive oxygen species (ROS), mitochondrial dysfunction, and by inducing inflammation [7].

The coculture of follicle shell-like GCs encapsulated in an agarose matrix with cumulus-oocyte complexes (COCs) indicated that GCs could improve oocyte developmental competence and blastocyst formation, reduce the intra-oocyte ROS content, and reduce apoptosis [8]. The study of Pawlak et al. [9] indicated that there is bidirectional cooperation of lipid metabolism between oocytes and surrounding cumulus cells. Besides, the coculture of porcine COCs with a monolayer of GCs reduced the stress induced by the presence of high level of NEFAs by activation of anti-inflammatory factors in GCs (unpublished data). However, the mechanisms of the positive effects of GCs on blastocysts obtained from cocultured oocytes exposed to high level of NEFAs are unclear.

*In vitro* studies on human follicles and embryos are limited because of ethical concerns and limitations. The pig is considered one of the optimal experimental models for human biomedical and reproductive research due to physiological, digestive, and reproductive similarities including the duration of oocyte *in vitro* maturation, the kinetics of cell division, and the timing of the embryo genome activation [10]. In this study, gene expression in porcine GCs cocultured with COCs and exposed to elevated level of NEFAs during IVM was analyzed to explore the pathways involved in protecting oocytes and improving blastocyst quality after *in vitro* fertilization.

## Results

### Differentially expressed genes

**Fig. 1:**
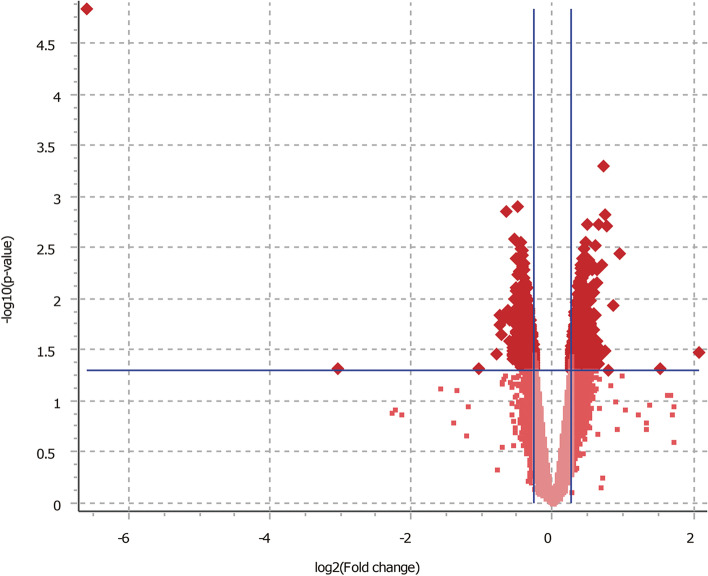
Volcano plot visualizing the statistical difference of the DEGs. The horizontal axis represents the *p*-value of detected transcripts, and the horizontal blue line at –log10 (P-value) of 1.3 is the threshold of 0.05. The vertical axis represents the fold-change (FC) of detected transcripts and the vertical blue lines are the cut-off criterion of 1.2. Each data point represents a gene or a variant of a gene. The upregulated and downregulated genes in the NEFAs treated group are illustrated as the dark red spots upper right and upper left, respectively

Following loess and quantile normalization and the limma algorithm with Flexaray, 1,381 out of the 43,794 probes were differentially expressed in cocultured GCs with NEFAs relative to the control (P<0.05). Of these, 1,295 probes were differentially expressed with the cut off criterion of 1.2-fold change (Fig. 1), and only 39 probes were differentially expressed with the cut off criterion of 1.5-fold change. With the verification of the database, 1,013 probes were annotated as differentially expressed genes and 438 of them were significantly upregulated in NEFA exposed GCs compared to the non-NEFA group, and 575 were downregulated.

### RT-qPCR

**Fig. 2:**
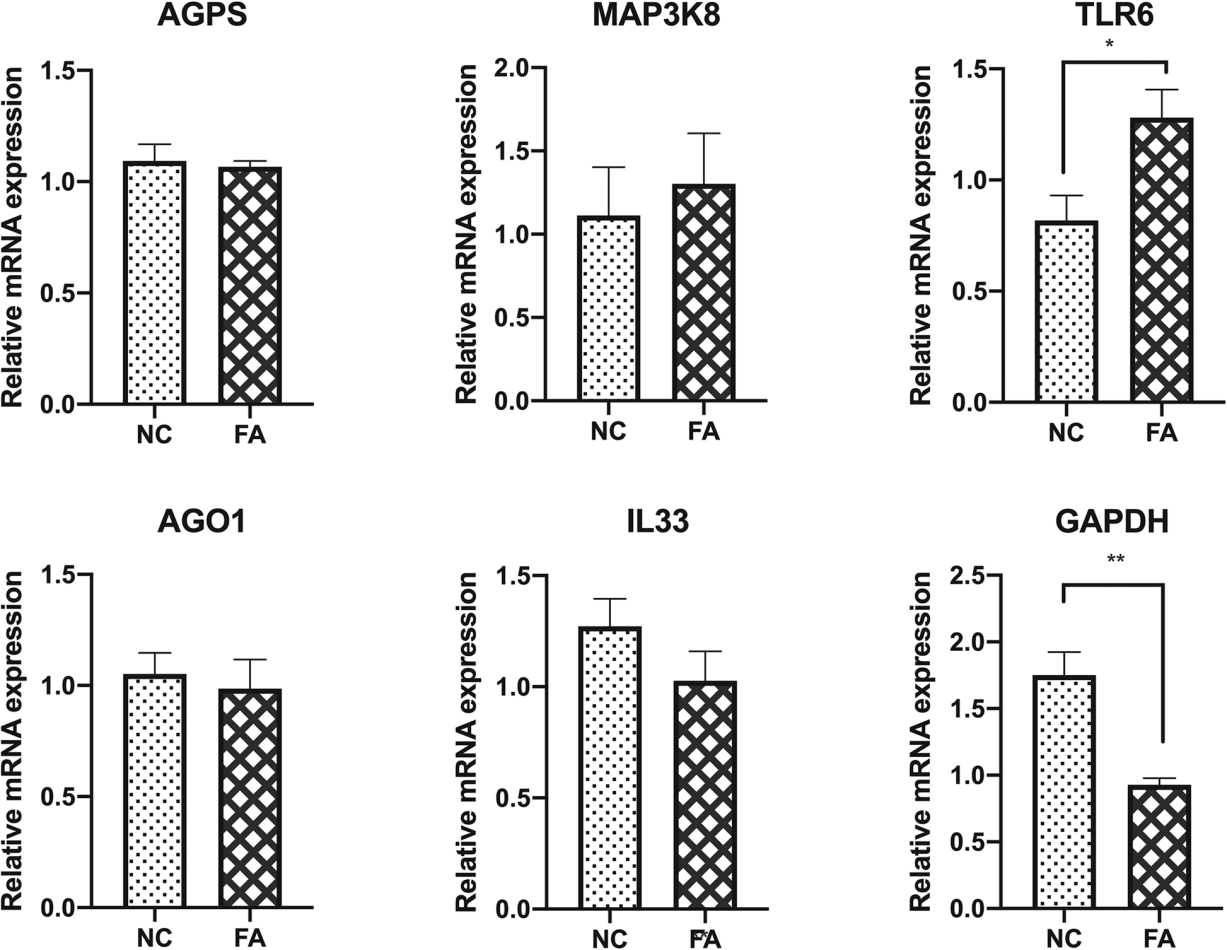
The abundance of different transcripts from genes affected by the elevated level of NEFAs (upregulated: AGPS, MAP3K8, and TLR6 and downregulated: AGO1, IL33, and GAPDH) was analyzed by real-time RT-qPCR. The RT-qPCR results showed that MAP3K8, TLR6, AGO1, IL33, and GAPDH had the same direction as in the microarray data, and two of them (TLR6 and GAPDH) showed significant differences relative to the respective controls. * represents p-value < 0.05, ** represents p-value < 0.01. GC samples were analyzed after IVM under the high level of NEFAs throughout the IVM culture or control treatment. NC is the control group, and FA is the group exposed to the elevated level of NEFAs. AGPS: Alkylglycerone Phosphate Synthase; MAP3K8: Mitogen-Activated Protein Kinase Kinase Kinase 8; TLR6: Toll Like Receptor 6; AGO1: Argonaute RISC Component 1; IL33: Interleukin 33; GAPDH: Glyceraldehyde-3-Phosphate Dehydrogenase

The other four granulosa cell replicates collected were used for SYBR green-based qRT-PCR to validate the expression level of six DEGs identified by microarrays. We observed a significant correlation between the qRT-PCR results and the microarray data for the six DEGs. The expression of Mitogen-Activated Protein Kinase Kinase Kinase 8 (*MAP3K8)*, Toll Like Receptor 6 (*TLR6)*, Interleukin 33 (*IL33)*, and Glyceraldehyde-3-Phosphate Dehydrogenase (*GAPDH)* had similar trends, and *TLR6* and *GAPDH* were significantly expressed by both methods (P<0.05), confirming the reliability of the microarray analysis (Fig. 2).

**Fig. 3:**
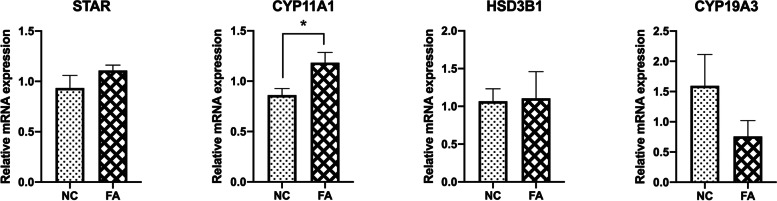
Expression of genes related to steroidogenesis. GCs were cultured for 7 days to form a monolayer. On day 8, a monolayer of GCs was cocultured with COCs either with or without the supplementation with the high level of NEFAs during in vitro maturation. The transcript abundance of different genes related to estradiol and progesterone was determined after IVM. The expressions of STAR, HSD3B1, and CYP19A3 was not affected by the high level of NEFAs. NC is the control group, and FA is the group exposed to the elevated level of NEFA. STAR: Steroidogenic Acute Regulatory Protein; CYP11A1: Cytochrome P450 Family 11 Subfamily A Member 1; HSD3B1: Hydroxy-Delta-5-Steroid Dehydrogenase, 3 Beta- And Steroid Delta-Isomerase 1; CYP19A3: Cytochrome P450 Family 19 Subfamily A 3

The expression of genes related to progesterone and estradiol production was also investigated. There was no significant change in the expression of Steroidogenic Acute Regulatory Protein [11], Hydroxy-Delta-5-Steroid Dehydrogenase, 3 Beta- And Steroid Delta-Isomerase 1 (*HSD3B1)*, and Cytochrome P450 Family 19 Subfamily A Member 3 (*CYP19A3*) in response to exposure to excessive NEFAs, and only the abundance of Cytochrome P450 Family 11 Subfamily A Member 1 (*CYP11A1)* was increased (Fig. 3) (P<0.05).

### Gene Ontology (GO) analysis and Kyoto Encyclopedia of Genes and Genomes (KEGG) pathways

**Fig. 4:**
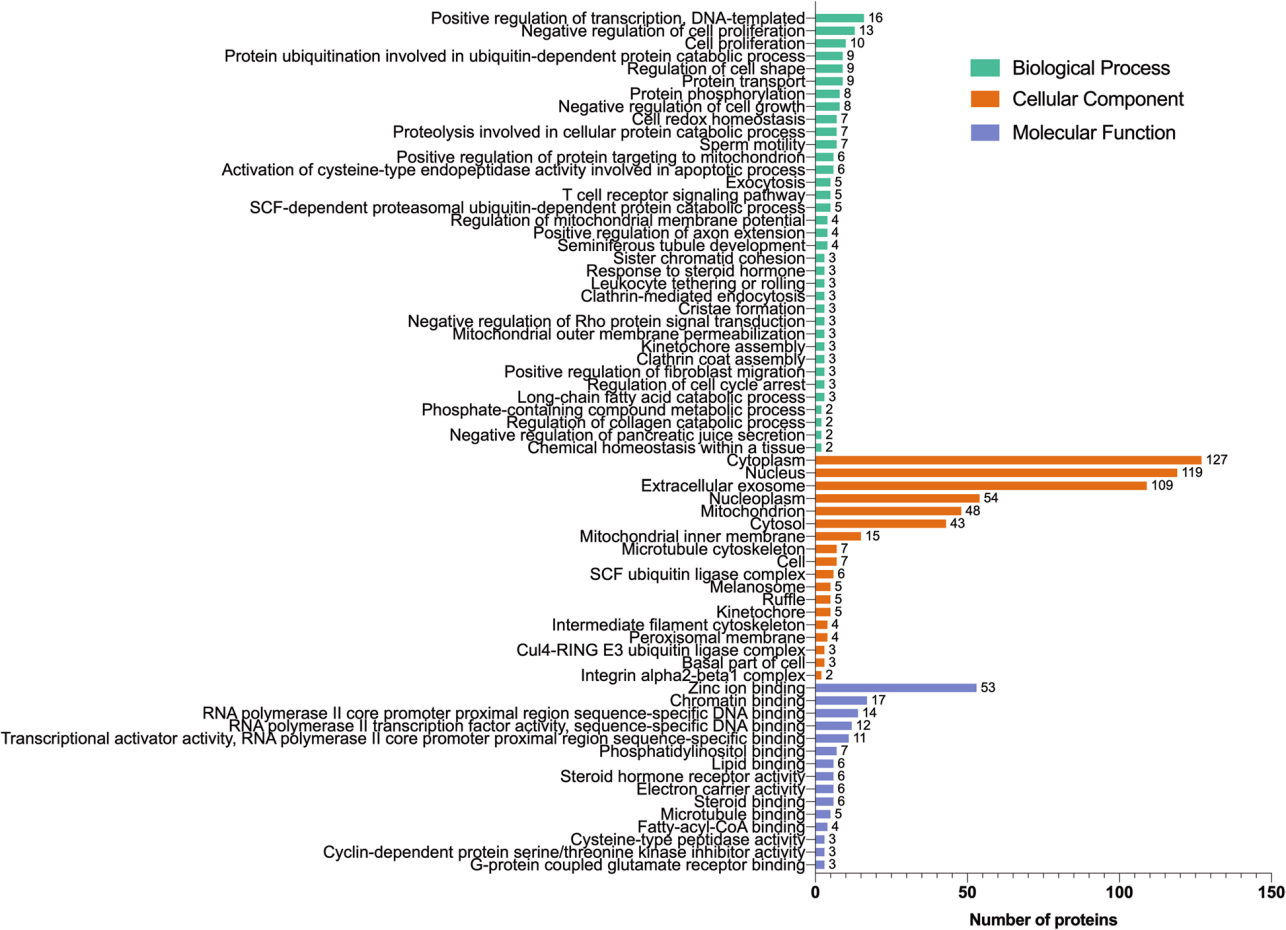
Gene ontology (GO) analysis was performed using DEGs between cocultured granulosa cells supplemented or not with NEFAs. The 2D bar graph contains biological process (green), cellular component (orange) and molecular function (purple) categories. The vertical axis shows terms of GO categories, and the horizontal axis shows the number of DEGs

To further explore the biological functions associated with the differentially expressed genes, GO annotations were applied. With the annotation of DAVID, 827 IDs were recognized and categorized into 35 biological processes, 18 cellular components, and 15 molecular functions (Fig. 4)(Supplemental Table S1). In terms of percentage of associated genes (one of the enrichment factors), the top significantly enriched biological processes were transcription-related regulation, cellular processes including cell proliferation, growth and cell cycle, as well as localization, catabolism, and protein modification. Interestingly, cell redox hemostasis and apoptosis were also biological processes affected by the high level of NEFAs. In the category cellular components, genes were clustered into terms related to integrin complex, ubiquitin ligase complex, and intracellular structures. The dominant molecular functions were binding of zinc, chromatin and lipid, and transcription regulation.

**Fig. 5:**
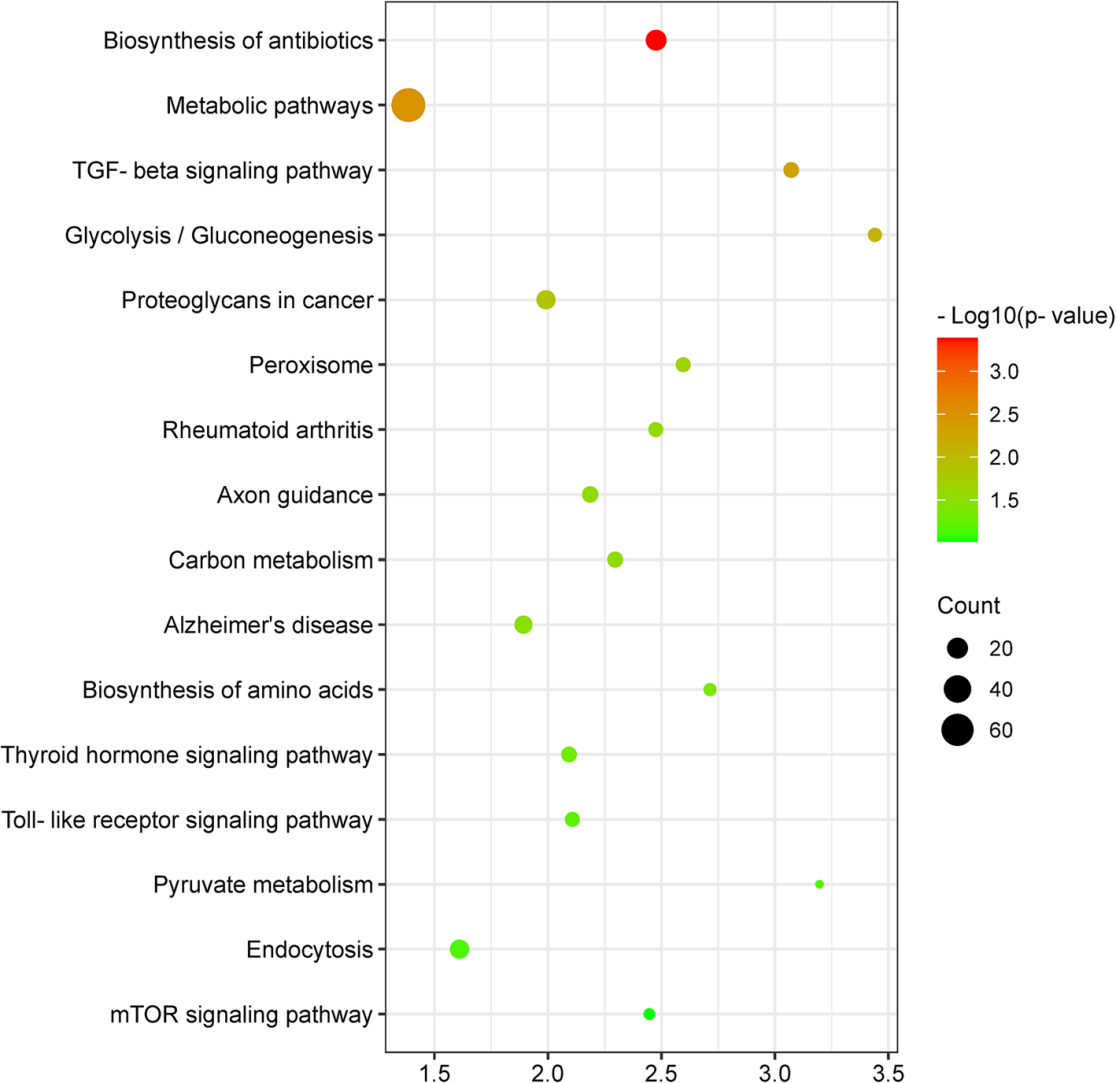
Bubble chart of potential signaling pathways generated by KEGG. Pathway analysis was performed to associate the unique DEGs with pathways using the KEGG database. The size and color of each bubble represent the number of DEGs in the pathway and the *P*-value, respectively. Fold enrichment was calculated as the ratio of genes (the number of genes from the dataset enriched in the pathway in question divided by all the genes from the dataset) divided by the ratio of background (the number of annotated genes from the background enriched in the pathway in question divided by all annotated genes from background)

The KEGG analysis showed that differentially expressed genes were markedly enriched for [1] metabolism, including carbohydrate, amino acid, and antibiotics; [2] inflammatory responses, including “Toll-like receptor signaling pathway” and “Rheumatoid arthritis”; [3] signal transduction, including “TGF-beta signaling pathway”, “mTOR signaling pathway” and “Thyroid hormone signaling pathway”; and [4] catabolism, including “Peroxisome” and “Endocytosis” (Fig. 5).

### IPA canonical pathway, tox list, and upstream regulators

**Fig. 6:**
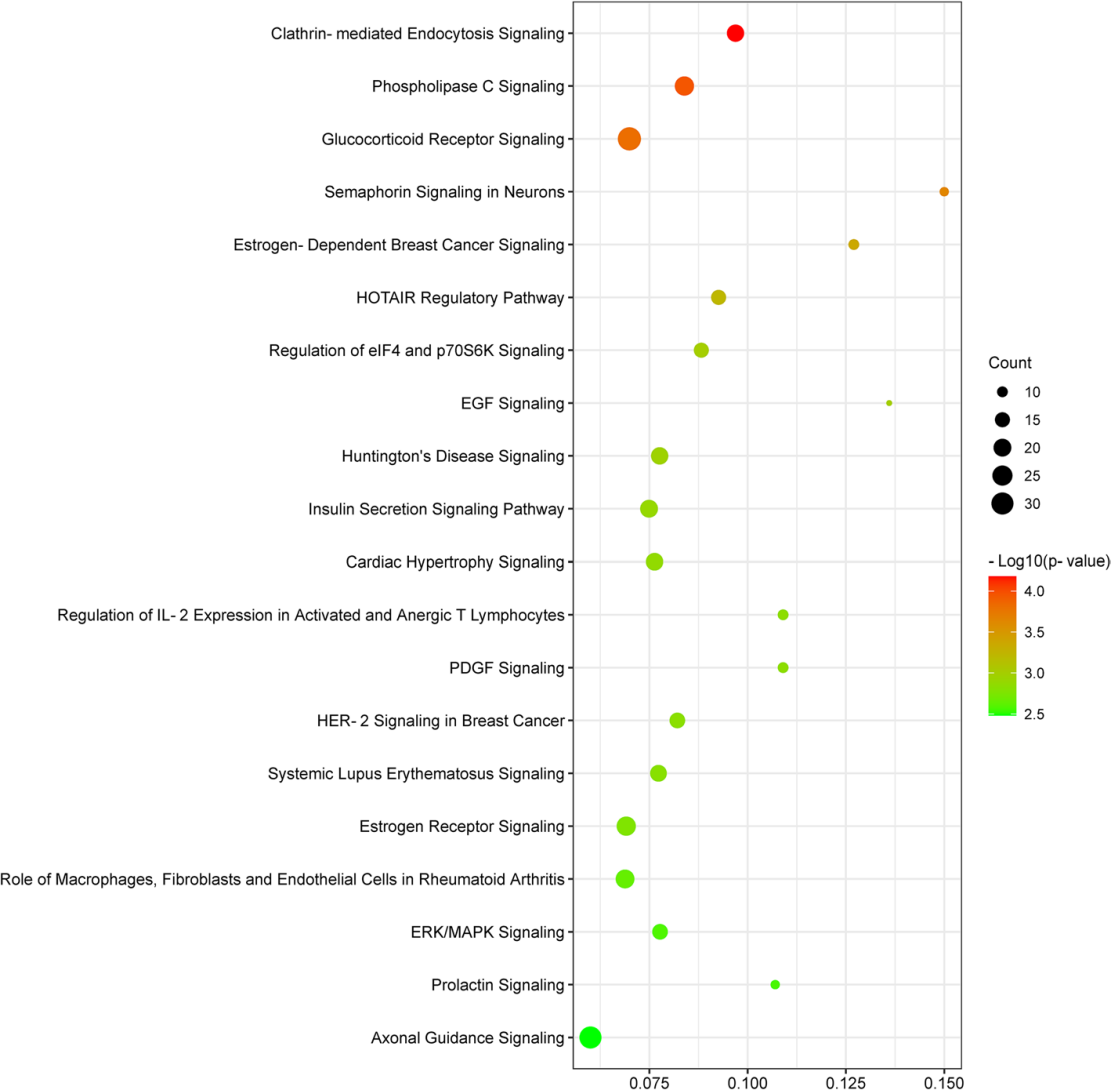
Bubble chart of the top 20 canonical pathways generated by IPA analysis. The size and color of each bubble represent the number of DEGs in each pathway and the *P*-value, respectively. The ratio was calculated as the number of genes from our dataset that overlap with the canonical pathway in question divided by the total number of genes that are represented in that canonical pathway

**Fig. 7:**
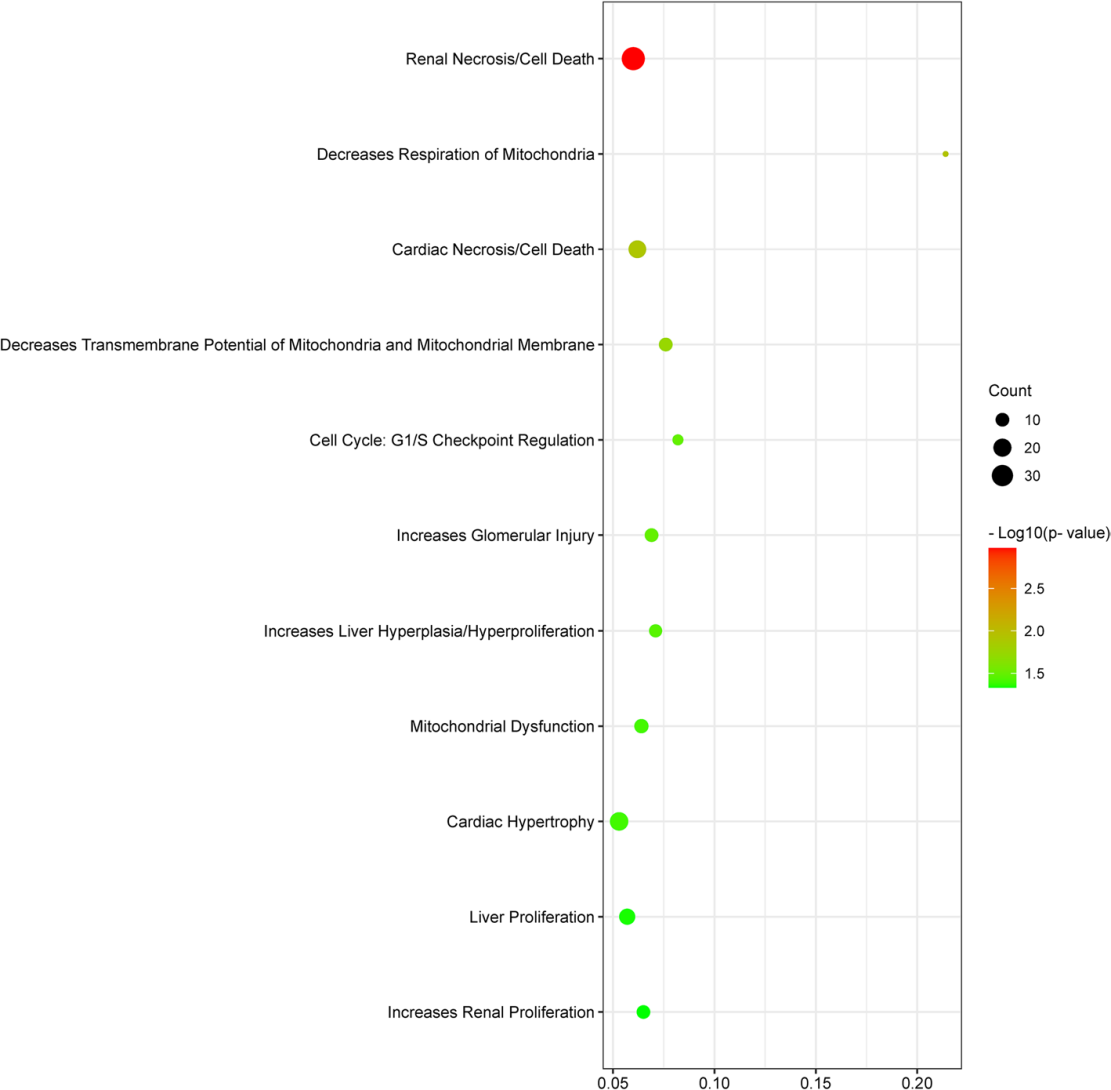
Bubble chart of tox lists affected by the high level of NEFAs with a p-value less than 0.05, which was generated by IPA analysis. The size and color of each bubble represent the number of DEGs in each pathway and the *P*-value, respectively. The ratio was calculated as the number of genes of our dataset that overlap with the tox list divided by the total number of genes that are represented in that tox list

**Fig. 8:**
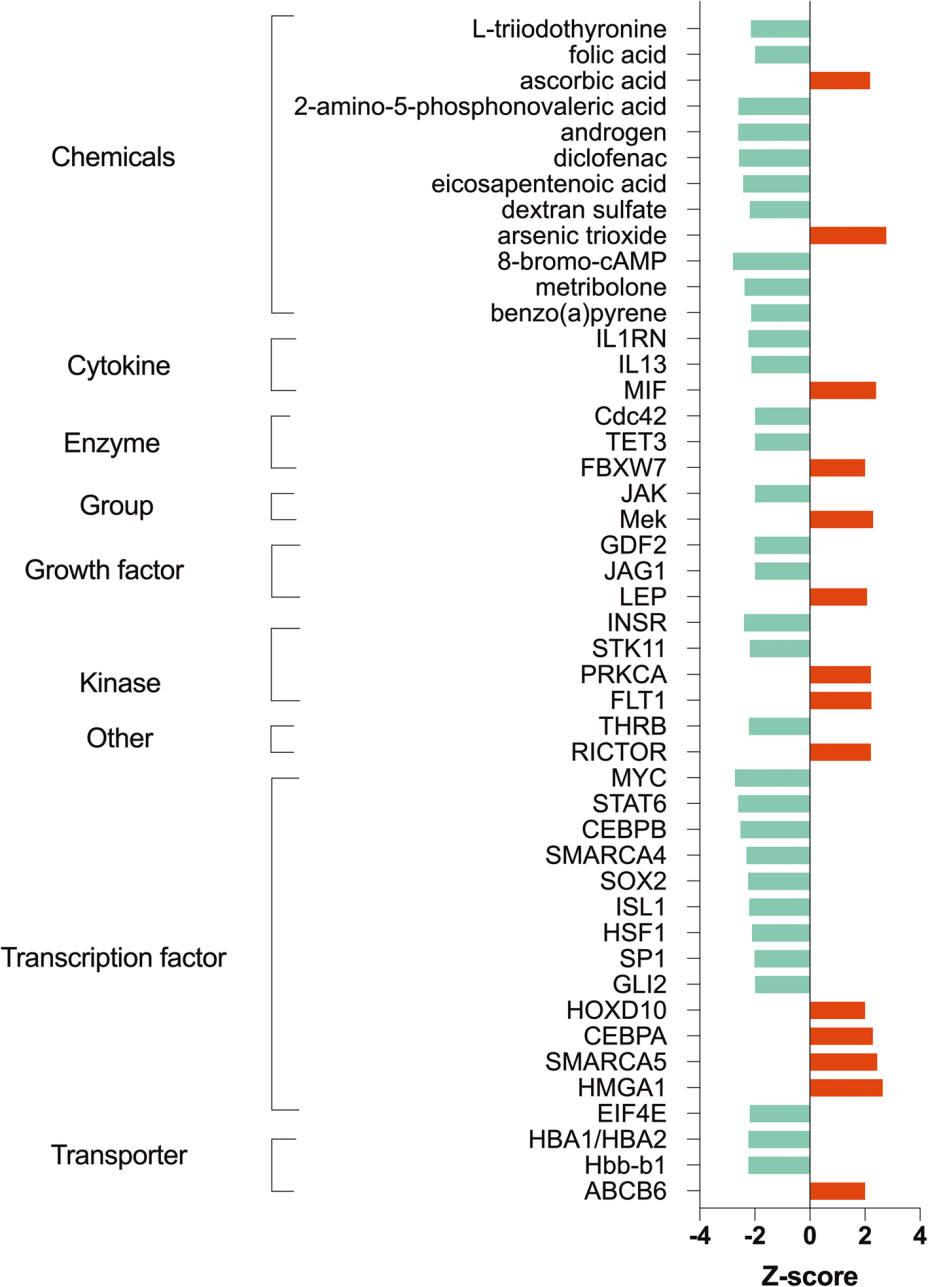
Upstream regulators with predicted activation or inhibition (with an absolute value of Z-sore greater than 2) affected by the high level of NEFAs from IPA analysis. These upstream regulators were categorized into 9 types including chemicals, cytokine, enzyme, group, growth factor, kinase, other, transcription factor, and transporter. The vertical axis shows categories and names of regulators, and the horizontal axis shows the Z-score (red represents activated, green represents inhibited)

Ingenuity Pathway Analysis with human, mouse, and rat data only recognized 876 probes, including 374 up-regulated and 502 down-regulated (Supplemental Table S2). The pathways most affected by the NEFA treatment (pathways which had low *p*-values) were “Clathrin-mediated endocytosis signaling”, “phospholipase C signaling”, “glucocorticoid receptor signaling”, “semaphoring signaling in neurons”, “estrogen-dependent breast cancer signaling”, “HOTAIR regulatory pathway”, “regulation of eIF4 and p70S6K signaling”, “EGF signaling”, “Huntington’s disease signaling”, and “Insulin secretion signaling pathway” (Fig. 6) (Supplemental Table S3). These highly significant pathways are related to endocytosis, signaling transduction, and metabolism, in agreement with the results from the KEGG database analysis. Besides, differentially expressed genes affected by excessive NEFA exposure were enriched in the tox lists related to “cell death” and “mitochondrial dysfunction” including “decreases respiration of mitochondria” and “decreases transmembrane potential of mitochondria and mitochondrial membrane” (Fig. 7) (Supplemental Table S4). The most significant upstream regulators identified were beta-estradiol, erb-B2 receptor tyrosine kinase 2 (ERBB2), trichostatin A, tumor protein P53 (TP53), and dexamethasone (Supplemental Table S5) Fig. 8.

## Discussion

Elevated levels of NEFAs are caused by either lipolysis or fat intake; therefore, the concentration of NEFAs varies importantly according to body condition and nutrition. Elevated NEFAs activated ROS-mediated inflammation, apoptosis, mitochondrial dysfunction, and insulin resistance in hepatocytes [12–14]. Moreover, high levels of NEFAs reduced reproductive performance in humans and bovine [1, 15]. High levels of NEFAs also induced apoptosis and impaired cell viability, and steroidogenic capacity in cultured ovarian cells [3–5]. Moreover, cumulus cells were shown to protect oocytes from the stress of excess NEFAs and to allow better blastocysts to be generated [16, 17]. In accordance with this, our previous study found that blastocysts which originated from oocytes exposed to elevated NEFA levels during IVM had impaired mitochondrial functions and increased inflammatory response [7] (unpublished data). Besides, some of the affected pathways in blastocysts from oocytes cocultured with GCs during IVM were associated with reduction of inflammation, indicating that the GCs were protecting the cocultured COCs and especially the oocytes (unpublished data). In this study, the original results provided some explanation on how fatty acids may rapidly impact the functions of GCs and how the cells adapt to their presence by triggering a defense mechanism.

### Inhibited luteinization of cocultured GCs under elevated NEFAs levels

Granulosa cells are one of the epithelial cell types that display polarity, cellular adhesion, and cell-cell junctions. Following the LH surge, GCs lose their epithelial nature and undergo an epithelial-mesenchymal transition (EMT) known as “luteinization” [18–20]. Luteinization of GCs can be induced by physiological doses of LH, FSH, or 3’,5’-cAMP *in vitro* [21]. In this study, hepatocyte growth factor (HGF), EGF, and transforming growth factor-beta (TGFβ) signaling pathways were significantly affected by the elevated NEFA level (showed in IPA canonical pathways and KEGG pathways), and these factors are involved in the initiation and regulation of the EMT [20, 22]. Moreover, IPA analysis results suggested that the EMT process was downregulated in GCs exposed to high NEFAs concentration. The MYC proto-oncogene (MYC), specificity protein 1 (SP1), and jagged canonical notch ligand 1 (JAG1) are involved in inducing the EMT [23–25]. Prediction of their down-regulation in this study implies that the EMT from GCs to luteal cells was possibly inhibited *in vitro* in the presence of COCs and NEFAs. A recent study suggested that increased concentrations of oleic acid (OA) may promote the EMT process in bovine GCs [26]. Oleic acid was demonstrated to have protective effects against lipotoxicity in oocytes and early embryos, contrarily to palmitic acid (PA), stearic acid (SA), and probably NEFAs (a combination of PA, SA, and OA)[9, 27, 28]. The promotion of luteinization by OA partly supports our result that the high level of NEFAs inhibited luteinization to some extent. The inhibition of EMT is somewhat similar to what was observed in post-partum dairy cows where high NEFAs were associated with slow follicular growth and decreased differentiation leading to delayed ovulation and infertility [29, 30]. Besides, elevated levels of NEFAs affected follicle development and inhibited ovulation in bovine [31]. In humans, extreme obesity is associated with amenorrhea and polycystic ovary syndrome, and both conditions can lead to impairment of the final differentiation of the follicle prior to ovulation [32, 33]. Moreover, the coculture of COCs may also interfere with the EMT transition of GCs because the maintenance of efficient bidirectional interaction between granulosa cells and COCs can reduce apoptosis in GCs [34].

Moreover, elevated NEFA levels were also reported to decrease progesterone production in bovine thecal cells, and inhibit testosterone production in mouse Leydig cells [35, 36]. These results can be explained by the inhibitory effect of NEFAs on the hydroxylation of cholesterol [36]. Besides, increased OA concentrations decreased the production of estradiol in bovine GCs [26]. In the present study, the expression of *HSD3B1*, which is involved in the conversion from pregnenolone to progesterone, was not affected by the elevated level of NEFAs, suggesting that progesterone production was not increased. The protein STAR is responsible for the transportation of cholesterol, the substrate for steroidogenesis; and *CYP11A1* encodes P450scc, which catalyzes the formation of pregnenolone, the precursor for both progesterone and estradiol [37]. In the pig genome, there are three paralogs (CYP19A1, CYP19A2, and CYP19A3) of the aromatase-encoding gene CYP19A, the key gene of estradiol production, and CYP19A3 is highly expressed in the ovary [38, 39]. The maintained and increased levels of these two genes indicate that cocultured GCs can maintain estradiol production which keeps the follicle in development, and that luteinization of cocultured GCs was not stimulated under the physiologically elevated NEFA levels used here. The contradictory results may be explained by the counteracting effects of OA against PA, SA, and NEFAs. Whether higher NEFA levels (either one saturated fatty acids or mixed fatty acids) would inhibit progesterone production or accelerate luteinization in pig GCs needs to be explored.

### Apoptosis and Inflammation

When cells are in the presence of an excess of NEFAs, these NEFAs are stored as lipid droplets, or they enter mitochondria to be β-oxidated to produce ATP. However, elevated NEFAs remaining in the cytosol may undergo lipid peroxidation, generating ROS and depleting protective glutathione levels [40]. The regulation of peroxisome, glycolysis/gluconeogenesis, and pyruvate metabolism highlighted in this study indicates that the original balance between β-oxidation and glycolysis in GCs was disrupted by the elevated NEFAs. Besides, the affected cell redox hemostasis suggests an increase in ROS production. In hepatocytes, NEFAs can activate oxidative stress-mediated NF-κB signaling to induce an inflammatory response [13]. In our previous study, exposure to elevated NEFAs level during *IVM* resulted in blastocysts with increased inflammation, apoptosis, and mitochondrial dysfunction associated with ROS production [7]. In line with this fact, endocytosis, rheumatoid arthritis, and toll-like receptor signaling pathways were significantly affected in GCs in the present study. Besides, NEFAs induced apoptosis and inhibited the proliferation of bovine and porcine GCs [5, 41], and directly caused ER stress and increased the intracellular Ca^2+^ level in oocytes [40]. The most significantly affected upstream regulators in this study, ERBB2 and TP53, indicated that elevated NEFAs induced inflammation and apoptosis in GCs similar to what we observed in blastocysts obtained from NEFA-exposed oocytes (unpublished data). Cells can adapt to the increased lipid stress to survive, but prolonged stress initiates inflammation and apoptosis [42]. This study showed that NEFAs induced inflammation and apoptosis in cocultured GCs.

### Impaired mitochondrial function

The mitochondrion is the principal organelle that produces ATP, and its well-organized functioning guarantees the efficiency of cellular processes. The increased ROS content due to the β-oxidation of excess fatty acids may cause mitochondrial dysfunction. A balance of pyruvate and fatty acid oxidation is required in healthy mammalian oocyte mitochondria to maintain a low level of ROS production [42]. Besides, the disrupted balance mentioned above was induced by the high level of NEFAs suggesting increased production of ROS. Excessive palmitic acid induced ROS formation, caspase 3 activated apoptosis, and deterioration of mitochondria, which contributed to the degeneration of the cumulus cell layers [43]. In oocytes, NEFAs can induce oocyte mitochondrial dysfunction with abnormal ATP production and high ROS content [41, 44, 45]. Moreover, our previous studies also demonstrated NEFA induced mitochondrial dysfunction in blastocysts from oocytes exposed to NEFAs [7]. Consistent with the NEFA induced effects, mitochondrial functions and cell redox hemostasis were also affected in this study, suggesting that the oxidation of excessive NEFAs created more ROS and impaired the mitochondrial function in cocultured GCs.

### GCs protect COCs against exposure to excessive NEFA levels

There is a possible protection mechanism in follicular cells that can desaturate the potentially toxic saturated fatty acids into mono-unsaturated fatty acids [17]. Instead of a lipotoxic impact, unsaturated fatty acids generally have a moderate impact on somatic cells [46]. Lipid storage and the β-oxidation of NEFAs in GCs [40] may be able to partly explain how the GCs protected the cocultured COCs and especially the oocytes. The genes STAT6, MYC, SP1 were affected potential upstream regulators which are involved in inflammation, apoptosis, and ER stress [47, 48]. Downregulation of these regulators implies that the inflammatory and apoptotic responses, which should have been induced by elevated NEFAs, were attenuated to a certain degree in cocultured GCs. When we compared blastocysts obtained from oocytes exposed to NEFAs alone to blastocysts from oocytes exposed to NEFAs in the presence of GCs, we observed that the co-culture reduced the inflammation reaction. However, the precise factors involved remain unknown and more research is needed.

### Effects of the high levels of NEFAs on different ovarian cell types

When simultaneously exposed to elevated levels of NEFAs either *in vivo* (pathological nutrition status including NEB and obesity) or *in vitro*, the different ovarian cell types respond differently. Commonly, apoptosis is induced by elevated levels of NEFAs in pig, human, bovine, and mouse granulosa cells [4, 49, 50], and apoptosis and inflammation are also increased in blastocysts originated from oocytes exposed to NEFAs [2, 7, 51]. Since granulosa cells and blastocysts go through mitosis, the proliferation of granulosa cells and the cell number of blastocysts are also affected by high levels of NEFAs [35, 41, 51, 52]. Lipid content is increased in granulosa cells, cumulus cells, and oocytes [16, 41, 44, 53, 54], and mitochondrial dysfunctions are induced in granulosa cells, cumulus cells, oocytes, and blastocysts [42–44, 55, 56]. The cellular metabolism of granulosa cells and blastocysts originated from oocytes exposed to NEFAs is also changed because fatty acids can be oxidized and produce ATP [2, 7, 51]. The more specific responses to elevated levels of NEFAs are more dependent on the type of cells, including the development ability of oocytes and blastocysts (maturation, fertilization, and development), the steroidogenesis activity of granulosa cells and theca cells, and the epithelial-mesenchymal transition of granulosa cells to luteal cells [2, 5, 7, 9, 27, 28, 35, 52, 57, 58].

## Conclusions

**Fig. 9:**
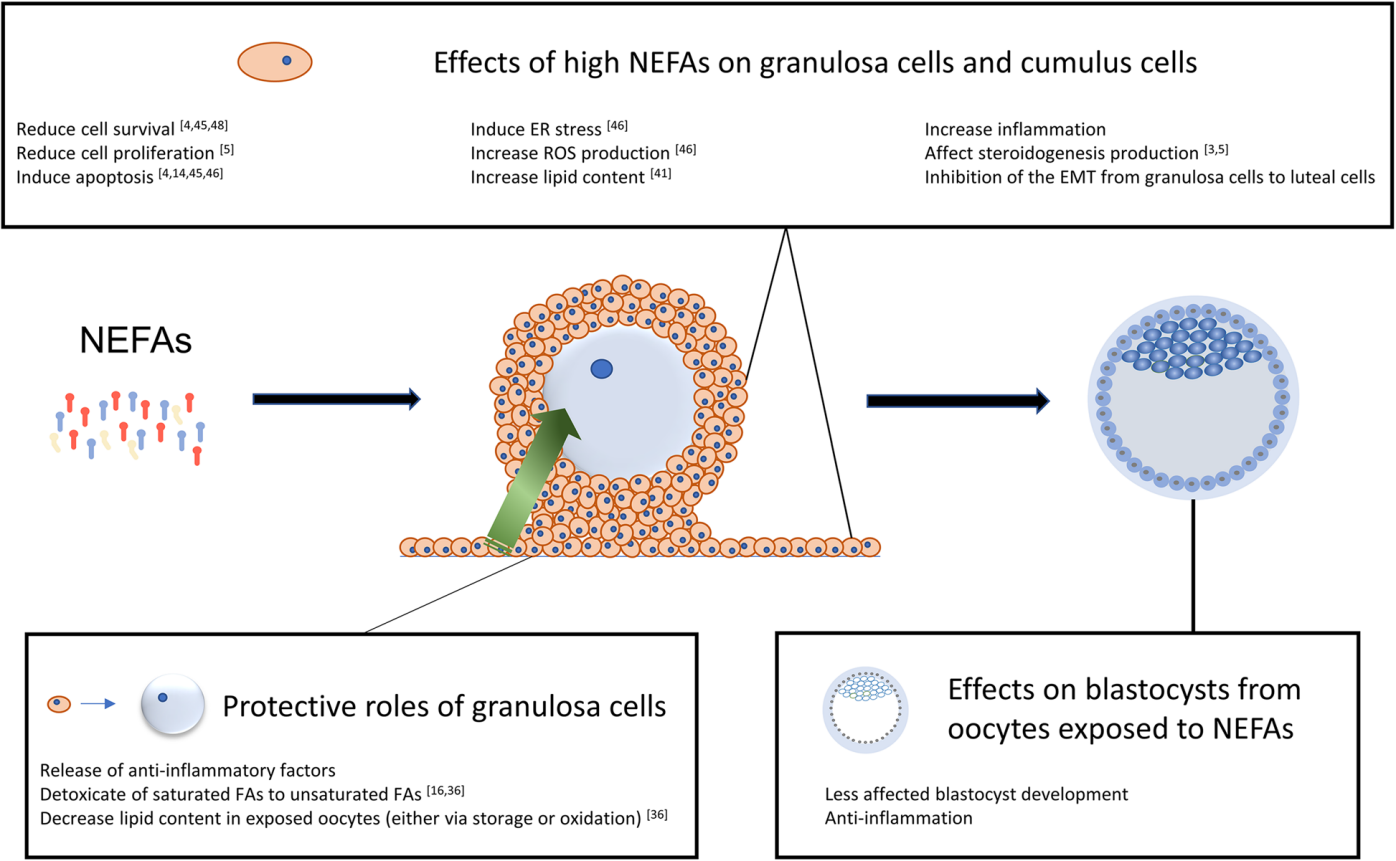
The response of GCs to high levels of NEFAs and how GCs protect COCs exposed to high levels of NEFAs. The cell survival, proliferation, and steroidogenesis of granulosa cells are affected by exposure to high levels of NEFAs. Apoptosis, ER stress, inflammation, and lipid accumulation in granulosa cells are increased. The epithelial-mesenchymal transition of granulosa cells to luteal cells is also negatively affected. Because the bidirectional communication between granulosa cells, cumulus cells, and oocytes ensures follicle development and oocyte maturation, granulosa cells and cumulus cells protect the oocyte when they are exposed to hazardous environments. The quantity of NEFAs that the oocyte is exposed to is reduced because some of the NEFAs are either stored as lipid droplets or oxidized in granulosa cells and cumulus cells. Besides, granulosa cells can produce and deliver some anti-inflammatory factors to cumulus cells and the oocyte. Therefore, blastocysts from oocytes exposed to the high level of NEFAs still exhibit the anti-inflammatory response, and the blastocyst rate from oocytes cocultured with GCs while exposed to NEFAs was decreased to a lesser extent than the blastocyst rate from exposed oocytes cultured without granulosa cells

The model for the mechanisms of the effects of high levels of NEFAs on GCs and how the GCs protect COCs is shown in Fig. 9. High levels of NEFAs in the IVM medium may inhibit the luteinization of porcine GCs cocultured with COCs, potentially explaining some delayed ovulation problems in other species. Besides, elevated NEFA levels increased the ROS-mediated inflammatory response in GCs, but this reaction may protect oocytes and explain the protective effect of GCs during IVM as described before.

## Materials and methods

### Chemicals

All supplements and reagents used in this study were purchased from sigma-Aldrich (St. Louis, MO, United State) unless otherwise specified.

### Preparation of NEFAs

PA, SA, and OA were dissolved in absolute ethanol at 468 mM, 194mM, and 534mM, respectively to make the stock solution as described previously [7]. The NEFA mixture was added to North Carolina State University (NCSU)-23 medium with 3 % fatty-acid-free bovine serum albumin and was sonicated for 30 min with an Arrayit ultrasonic bath sonicator (Sunnyvale, CA). The medium containing the NEFAs was then incubated at 38.5 °C in a humidified atmosphere of 5 % CO_2_ in air for 24 h for equilibration before use.

### Collection of GCs and COCs

Porcine ovaries were collected from a local slaughterhouse and transported within 2 h to the laboratory in physiological saline supplemented with 100 IU/L of penicillin G and 100 mg/mL of streptomycin sulfate at approximately 35 °C. The ovaries were washed three times with saline warmed to 37 °C and COCs, GCs, and follicular fluid were aspirated from follicles 3-6 mm in diameter with an 18-gauge needle attached to a 10-ml syringe, avoiding blood contamination. The cell mixture was washed twice in warmed Tyrode’s lactate-buffered HEPES supplemented with 0.1 % polyvinyl alcohol (PVA-TL-HEPES).

### Preparation of granulosa cell monolayers

After the COCs had fallen to the bottom of the container due to their larger size, the granulosa cell-containing superstratum of follicular fluid was passed through a Falcon 40-µm mesh cell strainer (BD Biosciences, Stockholm, Sweden), centrifuged at 800 × g for 8 min and washed three times with warm phosphate-buffered saline (PBS, Corning, New York, USA) containing penicillin-streptomycin (100 U per mL, ThermoFisher Scientific, Waltham, USA) and 50 µg/mL gentamycin. The cells were re-suspended at 5 × 10^5^ per mL in DMEM/F12 (ThermoFisher Scientific, Waltham, USA) supplemented with 10 % fetal calf serum (Corning, New York, USA), penicillin-streptomycin (100 U per mL) and 50 µg/mL gentamycin and cultured in 4-well plates (ThermoFisher Scientific, Waltham, USA) at 38.5 °C under a humidified atmosphere (5 % CO_2_). At the 24-h point, cells were washed with PBS twice and re-supplemented with fresh Dulbecco’s minimal essential medium/Ham’s F12 (DMEM/F12) with 10 % fetal calf serum for 6 more days of culture during which the medium (70 %) was changed every 48 h.

### Co-culture of COCs and granulosa cell monolayers

After 7 days of culture, the DMEM/F12 culture medium was replaced with NEFA-containing NCSU-23 (or NCSU-23 without NEFA but with same amount of BSA and ethanol as control) supplemented with 10 ng/mL epidermal growth factor (EGF), 0.5 g/mL follicle-stimulating hormone (FSH), 0.5 g/mL luteinizing hormone (LH), 1 mM dibutyryl-cyclic adenosine monophosphate, 1 % (v/v) purified porcine follicular fluid, and 50 µg/mL gentamycin. COCs were collected from the follicular fluid of a different batch of ovaries then washed with PVA-TL-HEPES. Those with cytoplasmic uniformity and at least three layers of cumulus cells fully surrounding the oocyte [59] were selected for maturation in 4-well plates containing granulosa cell monolayers. After 22 h, COCs were washed, put back in the same plate with fresh NEFA-containing NCSU-23 (or control NCSU-23) supplemented with only EGF and follicular fluid and incubated for another 22 h. After maturation, COCs were removed from the culture medium and the GC monolayers were washed three times with PBS. Then, Trizol (ThermoFisher Scientific) was used to capture the adherent cells and the mixture was stored at – 80 °C until used.

### RNA extraction

The Direct-zol^TM^ RNA Miniprep Plus (Zymo Research, CA, USA) was used to extract the RNA of GCs in Trizol. Cells from four wells of one 4-well plate were pooled as one replicate, and eight biological replicates were collected on different days (four for microarray analysis and four for quantitative real-time reverse transcription polymerase chain reaction (qRT-PCR) validation). An equal volume of ethanol was added to cells lysed in Trizol and mixed thoroughly. The mixture was passed through a Zymo-Spin^TM^ IIIGG column with a DNase I treatment, followed by the washing buffer. The quality and concentration of RNA were analyzed with a Bioanalyzer (Agilent, Mississauga, Ontario, Canada). All the extracted RNA samples were of good quality, with integrity numbers > 8.5. Samples were stored at −80 °C.

### Manipulation of RNA microarrays

Five ng of RNA from four replicates from two conditions were amplified linearly using a RiboAmp® HS PLUS RNA Kit (Applied Biosystems). Antisense RNA (aRNA) was produced using T7 RNA polymerase and labeled with Cy3 and Cy5 using a ULS Fluorescent Labeling Kit (Leica, Germany). Two corresponding treatments (900 ng aRNA per replicate) were mixed in a two-color dye-swap design and hybridized on an Agilent-manufactured EmbryoGENE 4 × 44 K porcine transcriptome microarray slide (Agilent Technologies, CA, USA) for 17 h at 65 °C. The slide was washed for 1 min in Expression Wash Buffer 1 (RT), 3 min in Expression Wash Buffer 2 (42 °C), 10 s in 100 % acetonitrile (RT), and 30 s in Stabilization and Drying Solution. Microarray slide was then scanned with the PowerScanner (Tecan, Switzerland) and features were extracted with Array-pro 6.3 (MediaCybernetics, MD). Intensity files were normalized and analyzed with FlexArray. The background was subtracted from raw fluorescence intensity prior to normalization within Cy-3/Cy-5 and between each array with Loess and quantile, respectively.

### Data analysis

Treatment effects were analyzed with the limma algorithm, which attributed to each probe a probability of differing significantly from the control. The differentially expressed genes (DEGs) were then annotated by the latest version (Revision 6) of the Affymetrix porcine annotation database combined with the current annotation data of pig (*Sus scrofa* 11.1) in NCBI [37]. GO and pathway enrichment analyses were performed using the DAVID Database (V6.8) with *Sus scrofa* background [60]. Data were also analyzed using Ingenuity Pathway Analysis (IPA, Ingenuity® Systems, www.ingenuity.com/) with human, mouse, and rat backgrounds. IPA analysis considers the general tendency in gene expression variations, including the direction in which each tends to change and the concordance between the variations of the significantly affected genes and the signaling pathways. It then determines the most plausible upstream regulators (explaining the observed gene expression changes) and tox list (linking to pharmacological responses) by taking into consideration the results and the usual context in which the same genes are affected. The extremely high similarity between the porcine and human genomes and the availability of more annotated functions in human studies made the IPA analysis a necessary complement.

### Quantitative Real-Time RT-qPCR

Total RNA extracted from the other four biological replicates was reverse transcribed using oligo-dT and random primers with the qScript^TM^ Flex cDNA Synthesis Kit (Quanta Biosciences, MD). Real-time PCR reactions were conducted on a LightCycler 480 System (Roche, Germany) with LightCycler 480® SYBR Green I Master. The efficiency of the primers and the standard curve for each gene were deduced from serial dilutions (1 pg to 0.1 fg) of the corresponding cDNA fragment used as template. The geometric mean of two housekeeping genes, succinate dehydrogenase complex flavoprotein subunit A (*SDHA*) and peptidylprolyl isomerase A (*PPIA*), [61], was used to normalize gene expression with the GeNorm VBA applet software. Primer information is provided in Table 1. Statistical analysis was conducted in Prism (GraphPad Software, version 5.00 for Windows, San Diego California USA) with the independent-samples t-test.

**Table 1:** Information on RT-qPCR primers

Target gene	Accession number	Forward primer (5’-3’)	Reverse primer (5’-3’)	Product size	Annealing Temperature
*AGPS*	XM_021075128.1	GACGCAGACTTATGATGCAGG	AGCCACTGCTTCCGTAACT	180 bp	57 °C
*MAP3K8*	XM_021064737.1	ATGGAACCGTGGAGGACTTG	CGGAGCCGATATTCCTGTAAGT	192 bp	57 °C
*TLR6*	NM_213760.2	CTCTCATGGCACAGCGAACT	CACATCATCCTCTTCAGCGACT	125 bp	59 °C
*AGO1*	XM_005665209.3	ACGCTGTTACCTCACTGGATAG	GGCCAGATGTGACAAGATGAGG	244 bp	59 °C
*IL33*	NM_001285978.1	ATGGTGGTGGTGATCATCGGA	ACACTCCAGGATGGCTCTTACA	201 bp	59 °C
*GAPDH*	NM_001206359.1	ACTGGTGTCTTCACGACCAT	GGAGGCATTGCTGACGATCT	159 bp	57 °C
*STAR*	NM_213755.2	CCCTTGGACAGGAGCTGAAC	GGCCAGATCTTGGTCACTGT	134 bp	57 °C
*CYP11A1*	NM_214227.1	AGACACTGAGACTCCACCCCA	GACGGCCACTTGTACCAATGT	110 bp	57 °C
*HSD3B1*	NM_001004049.2	GATCGTCCACTTGTTGCTGG	GATCTTGCTCTGGAGCTTAGAA	109 bp	57 °C
*CYP19A3*	NM_214431.1	GTGGACGTGTTGACCCTCAT	TATGCACGAGGGCACTTTCG	94 bp	57 °C
*SDHA *[61]	XM_021076930.1	CACACGCTTTCCTATGTCGATG	TGGCACAGTCAGCTTCATTC	94 bp	57 °C
*PPIA *[61]	NM_214353.1	AAAACTTCCGTGCTCTGAGC	TTATGGCGTGTGAAGTCACC	112 bp	59 °C

Note: The superscript number beside a target gene indicates the reference from which the primer sequence was obtained

## Supplementary Information

**Additional file 1.**

**Additional file 2.**

**Additional file 3.**

**Additional file 4.**

**Additional file 5.**

## Data Availability

The data sets of transcriptomic microarray results were deposited in National Center for Biotechnology Information’s Gene Expression Omnibus and are accessible through the accession number GSE168957.

## Abbreviations

NEFAs: Non-Esterified Fatty Acids
GCs: Granulosa Cells
COCs: Cumulus-Oocyte Complexes
IVM: In Vitro Maturation
ROS: Reactive Oxygen Species
ER: Endoplasmic Reticulum
PA: Palmitic Acid
SA: Stearic Acid
OA: Oleic Acid
NCSU: North Carolina State University
PVA-TL-HEPES: Tyrode’s Lactate-buffered HEPES supplemented with 0.1 % Polyvinyl Alcohol
PBS: Phosphate-Buffered Saline
DMEM/F12: Dulbecco’s Minimal Essential Medium/Ham’s F12
EGF: Epidermal Growth Factor
FSH: Follicle-Stimulating Hormone
LH: Luteinizing Hormone
aRNA: Antisense RNA
RT: Room Temperature
DEGs: Differentially Expressed Genes
GO: Gene Ontology
IPA: Ingenuity Pathway Analysis
SDHA: Succinate Dehydrogenase Complex Flavoprotein Subunit A
PPIA: Peptidylprolyl Isomerase A
qRT-PCR: Quantitative Real-time Reverse Transcription Polymerase Chain Reaction
MAP3K8: Mitogen-Activated Protein Kinase Kinase Kinase 8
TLR6: Toll Like Receptor 6
IL33: Interleukin 33
GAPDH: Glyceraldehyde-3-Phosphate Dehydrogenase
STAR: Steroidogenic Acute Regulatory Protein
HSD3B1: Hydroxy-Delta-5-Steroid Dehydrogenase, 3 Beta- And Steroid Delta-Isomerase 1
CYP11A1: Cytochrome P450 Family 11 Subfamily A Member 1
KEGG: Kyoto Encyclopedia of Genes and Genomes
ERBB2: Erb-B2 Receptor Tyrosine Kinase 2
TP53: Tumor Protein P53
EMT: Epithelial-Mesenchymal Transition
HGF: Hepatocyte Growth Factor
TGFβ: Transforming Growth Factor-beta
MYC: MYC Proto-oncogene
SP1: Specificity Protein 1
JAG1: Jagged Canonical Notch Ligand 1
